# High seroprevalence of feline morbilliviruses in free-roaming domestic cats in Chile

**DOI:** 10.1007/s00705-020-04882-2

**Published:** 2020-11-20

**Authors:** Johannes Busch, Irene Sacristán, Aitor Cevidanes, Javier Millán, Thomas W. Vahlenkamp, Constanza Napolitano, Michael Sieg

**Affiliations:** 1 Institute of Virology, Faculty of Veterinary Medicine, Leipzig University, An den Tierkliniken 29, 04103 Leipzig, Germany; 2 PhD Program in Conservation Medicine, Facultad de Ciencias de la Vida, Universidad Andres Bello, República 252, Santiago, Chile; 3 Facultad de Ciencias de la Vida, Universidad Andres Bello, República 252, Santiago, Chile; 4 Instituto Agroalimentario de Aragón-IA2 (Universidad de Zaragoza-CITA), Miguel Servet 177, 50013 Zaragoza, Spain; 5 Fundación ARAID, Avda. de Ranillas, 50018 Zaragoza, Spain; 6 Departamento de Ciencias Biológicas y Biodiversidad, Universidad de Los Lagos, Av. Fuchslocher 1305, Osorno, Chile; 7 Instituto de Ecología y Biodiversidad (IEB), Santiago, Chile

## Abstract

Feline morbillivirus infections have gained increased attention due to repeated reports of their association with urinary tract disease in cats. In the present study, 112 serum samples from free-roaming domestic cats in Chile were tested for antibodies against feline morbillivirus genotypes 1 and 2 (FeMV-1 and FeMV-2) using an indirect immunofluorescence assay. In total, 63% of the animals showed antibodies against one or both FeMV genotypes. Antibodies directed exclusively against FeMV-2 were significantly more prevalent in male cats. The correlation of sex and FeMV-2 infection might give insight into potential routes of transmission. We provide, for the first time, serological data on FeMV in Chile.

**Electronic supplementary material:**

The online version of this article (10.1007/s00705-020-04882-2) contains supplementary material, which is available to authorized users.

The family *Paramyxoviridae* currently comprises 78 virus species divided into four subfamilies and 17 genera covering a broad host range including mammals, birds, fish and reptiles [1]. In 2012, a new paramyxovirus was detected in stray cats from Hong Kong, designated as feline morbillivirus (FeMV, formally known as FmoPV) [2]. Subsequent studies verified FeMV to be present in Japan [3], Germany [4], Italy [5], the USA [6], Brazil [7], Turkey [8], the UK [9], Malaysia [10] and mainland China [11]. In-depth analysis of complete genome sequences revealed viral diversity of FeMV strains from different locations [3, 12, 13]. In 2019, a large surveillance program in Germany identified a second genotype of FeMV (FeMV-2) with 78% whole-genome nucleotide sequence identity to the previously detected FeMV isolates [14]. In infected cats, viral proteins were predominantly detected in the kidney but were also found in other organs (e.g., lymph nodes) [2, 12, 13, 15]. FeMV involvement in chronic kidney diseases (CKD) has been suggested. CKD is common in domestic cats, with a reported incidence of 28-50%, primarily affecting older animals [16, 17]. Prevalence data for FeMV obtained by detection of antibodies against the viral nucleoprotein (N) in Japan, Hong Kong and the UK revealed that 21.0, 27.8 and 30% of the animals, respectively, were FeMV positive, [2, 9, 12]. Similar results were obtained using a phosphoprotein (P)-based enzyme-linked immunosorbent assay (ELISA) [18]. In the USA and Brazil, FeMV has been detected by RT-PCR [6, 7], but so far, no serological studies have been published.

We analyzed serum samples from 112 domestic cats. The cohort comprised 62 female and 50 male, rural, free-roaming, mix-bred, short-haired cats, with an average age of 30 ± 4.2 months. Over 90% of the cats were less than 4 years of age. None of the animals had been neutered, vaccinated or dewormed. The sampling area comprised nine regions from central to southern Chile. In the present study, we determined the antibody status against FeMV-1 and FeMV-2 using an immunofluorescence assay (IFA) developed for both genotypes. Viruses were isolated from urine samples taken from two persistently infected cats in Germany. Propagation in cell culture was performed as described previously for FeMV-1 [19] and for FeMV-2 [14]. Whole genome sequences are available under GenBank accession no. MG563820 and MK182089, respectively. In brief, CrFK cells were infected with FeMV-1 (MOI = 0.01), and LLC-MK2 cells were infected with FeMV-2 (MOI = 0.01). After five days, cells were fixed with 80% acetone, blocked with 5% (w/v) BSA in PBS before cat sera were applied at a dilution of 1/100 (v/v) in 1% (w/v) BSA in PBS overnight at 4°C. After washing with PBS, a goat anti-cat IgG (H+L) Alexa Fluor 488–conjugated antibody (Dianova, Germany), diluted 1/500 (v/v) in 1% (w/v) BSA in PBS was applied for 30 min at 37°C. Prior to evaluation of the signal, cells were washed twice with PBS. Uninfected cells served as negative controls for each sample and allowed determination of virus-specific signals. Graphs as well as figures were generated using MS Office, and statistics were performed using the GraphPad QuickCalcs website to determine significance by two-tailed Fisher's exact test [20]. RT-PCR for the detection and further phylogenetic analysis of feline morbilliviruses was not possible due to the limited amount of serum available.

The IFA approach was evaluated using sera from the same persistently infected cats whose urine had been used to propagate the respective FeMV genotype, as well as specific antibodies against the FeMV N or P protein (shown in the supplementary file). Based on the observed fluorescence signals, cat sera were judged to be positive for FeMV-1 only, FeMV-2 only, positive for both genotypes (FeMV double positive) or FeMV negative. Representative results are shown in Figure 1, which shows viral intracytoplasmic inclusion bodies indicated by arrows. We found that 63% of the cats (71 animals) had antibodies against FeMV. Thirty percent of these samples were seropositive for both genotypes. It is currently unknown whether double-positive sera are the result of coinfections or consecutive infections, or possibly due to cross-reactive antibodies derived from either FeMV-1 or FeMV-2 monoinfection. Furthermore, 24% and 9% of the animals were positive FeMV-1 only and FeMV-2 only, respectively (Fig. 2). In combination with the staining pattern observed in Supplementary Figure 1, these data suggest that sera that are positive against only one genotype might be restricted in their response to either one viral protein or even a specific epitope. In addition, sex-related differences in seroprevalence of FeMV were investigated. As depicted in Figure 3, only slight differences between female (69%) and male (74%) cats were observed in the overall FeMV seroprevalence. However, statistically significant differences were detected between the sexes for the seroprevalence of FeMV-2 only (*p* = 0.0407). While 16% of male cats had antibodies against FeMV-2 only, just 3% of the female cats were seropositive for FeMV-2 only. Such a correlation was not observed for FeMV-1.

**Fig. 1:**
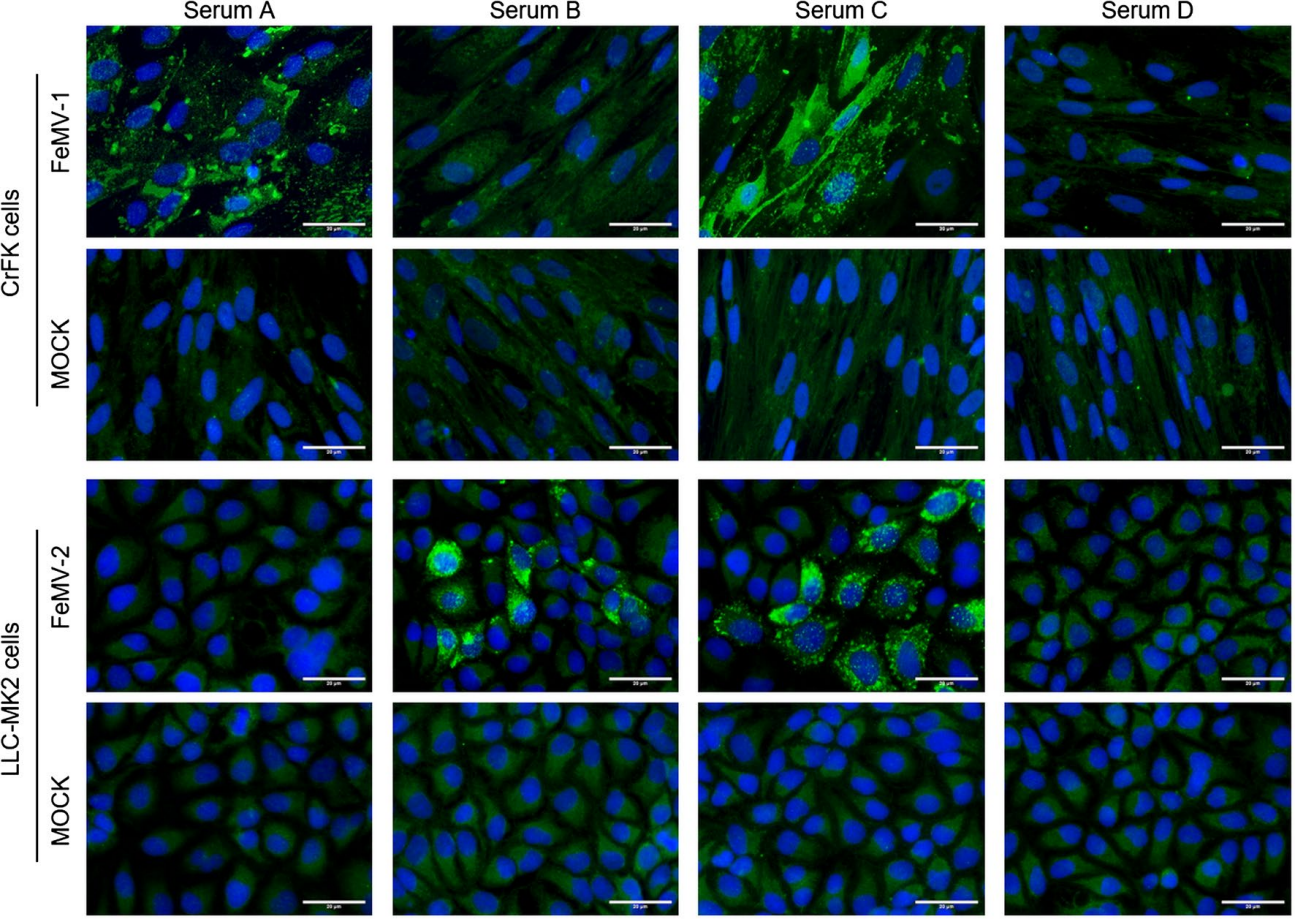
Representative images of IFA against both FeMV genotypes. Serum A was found to be positive for FeMV-1 only, serum B was positive for FeMV-2 only, serum C was positive for both types, and serum D was negative for both types. Arrows indicate virus-specific signals. Scale bars indicate 20 µm.

**Fig. 2:**
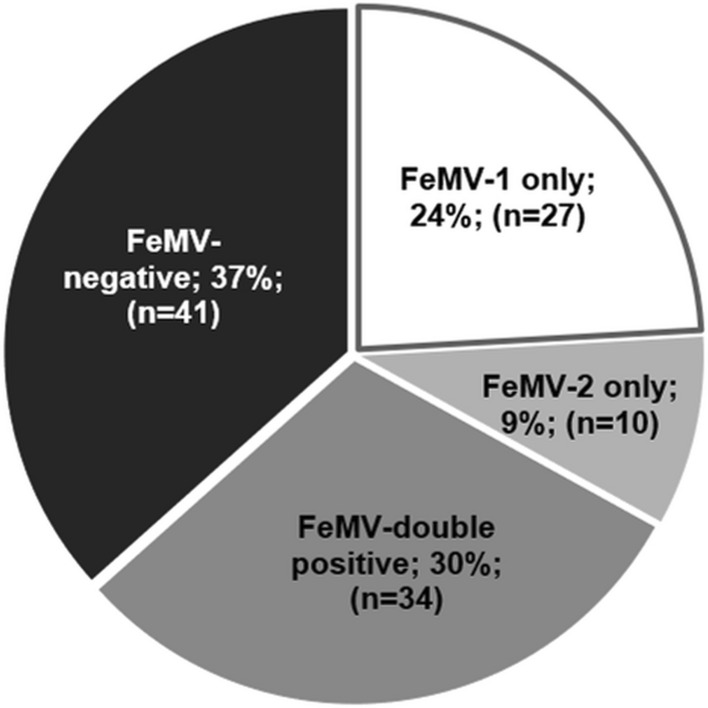
Seroprevalence of FeMV antibodies determined in samples from free-roaming domestic cats in Chile. Sera were tested separately for antibodies against each genotype

**Fig. 3:**
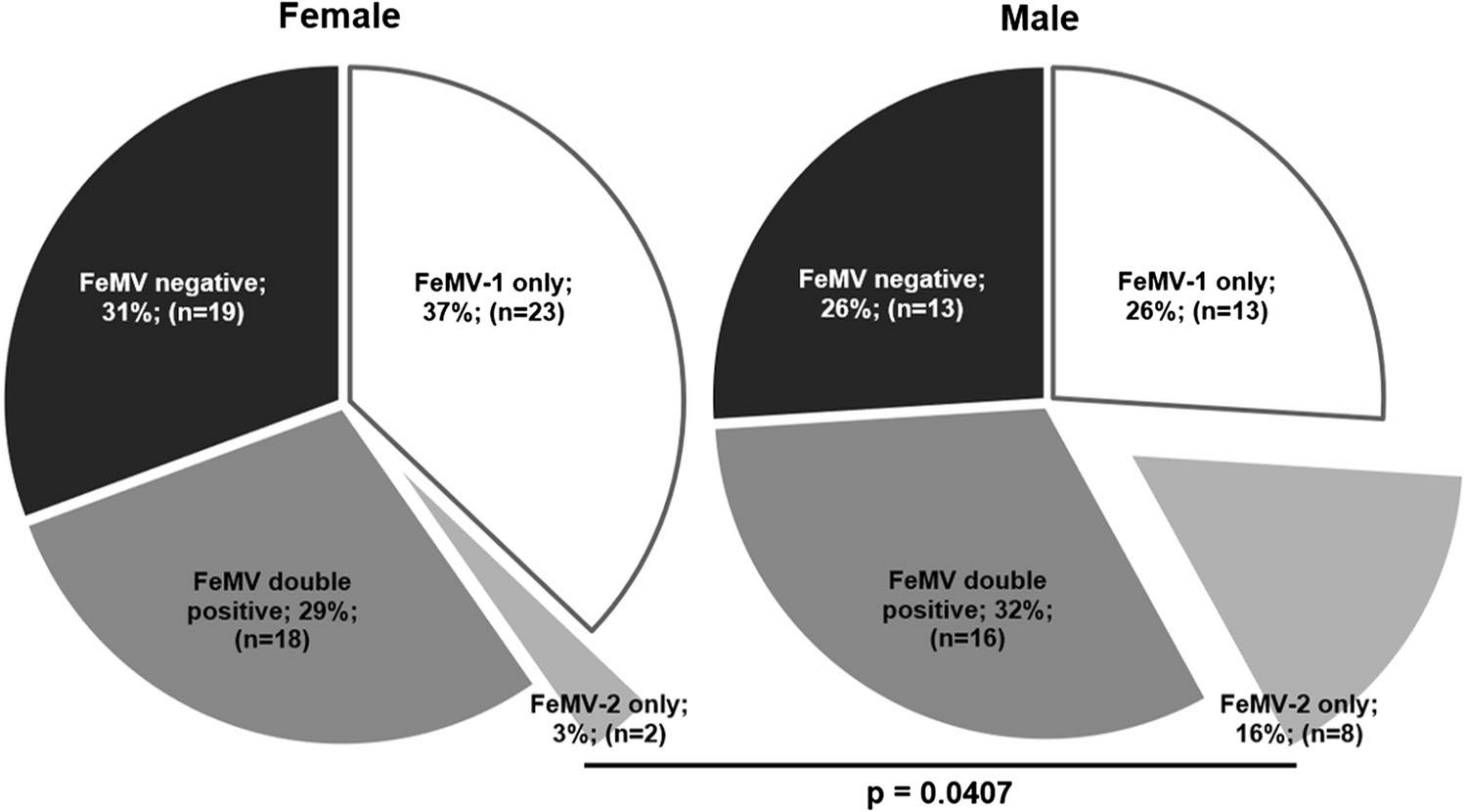
Comparison of seroprevalence rates of FeMV antibodies in samples taken from male and female cats in Chile. Statistical significance is shown as the *p-*value

Overall, our results are in accordance with previous studies using recombinant viral N protein in immunoblot assays [2, 9], N-protein-expressing cells [12], or recombinant-P-protein-based ELISA [18]. Our experimental setting allowed the detection of antibodies against all viral structural proteins simultaneously. This advantage might explain the higher seroprevalence detected in Chilean cats compared with studies from other countries. For instance, experiments conducted in Hong Kong, Japan, and the UK [2, 9, 12], used single-protein-based assays and thus might be unable to detect antibody responses to other viral proteins, e.g., the viral surface proteins. This is supported by a study using whole-virus immunoblot analysis [3]*,* which confirmed differences in antibody reactivity against structural FeMV proteins. The FeMV-specific antibody prevalence in Japanese cats was found to be 22%. Differences compared to our data (24% FeMV-1 only and additionally 30% FeMV double positive) might be due to the limited sample size (n = 13) in the previous study or country-specific differences in the epidemiological situation. Samples included in the study by Sakaguchi *et al.* published in 2014 were obtained from cats brought to a veterinary clinic for various reasons. Those cats are thus likely to have had an owner. In comparison, the cats in Chile analyzed in this study had an owner but were not confined and were in a rural setting (with the exception of one animal), and the likelihood of infection with FeMV might therefore have been higher. Another aspect that may explain the higher FeMV seroprevalence in Chile than in other countries might be the different global distribution of the two FeMV genotypes, since no serological data were obtained on the American continents, although FeMV RNA has been detected in the USA [6] and Brazil [7]. A complete genome sequence was available for the US strain, only. An amino acid sequence comparison between the FeMV-1 strain used in this study and the US strain (accession no. KR014147) revealed 97.11% sequence identity in the N protein, 86.73% in the P protein, 96.14% in the M protein, 94.29% in the F protein, 94.79% in the H protein, and 96.05% in the RNA polymerase protein. These data point towards a limited variability of FeMV-1 globally, and cross-reactivity of the antibodies generated can be assumed. A high degree of similarity among all available FeMV-1 strains was also shown previously [21]. The overall amino acid sequence identity in the N, P, M, F, H and RNA polymerase protein between FeMV-1 and FeMV-2 used in this study was found to be 90.94%, 77.19%, 91.39%, 89.50%, 86.53%, and 90.64%, respectively. Therefore, assays based solely on FeMV-1 sequences or proteins might underestimate the prevalence of FeMV-2. Using our IFA approach, we detected a significantly higher seroprevalence of FeMV-2 in male cats. This might be explained by the lack of neutering in this cohort, resulting in closer and more frequent social contacts between animals, increased roaming distance, urine spraying, and sexual activity [22, 23]. Like for FeMV-2, the seroprevalence of feline immunodeficiency virus (FIV) and feline leukemia virus (FeLV)' as well as 'canine distemper virus (CDV) have also been reported to be higher in male cats [24]. Since no such correlation was detected for FeMV-1, an alternative route of transmission may be considered.

Correlations with FeLV, FIV and **??**canine distemper virus**??** (CDV) data evaluated based on the samples of this investigation in the course of a previous study [25] showed that two cats that were double positive for FeMV-1 and FeMV-2 were also positive for FIV antibodies. All but one of the animals were negative for CDV antibodies [25]. Out of ten animals that were positive for FeLV antigen, two were positive for FeMV-1 only, two positive for were FeMV-2 only, and two were positive for both FeMV-1 and FeMV-2. Furthermore, four FeLV-antigen-positive cats were FeMV negative. Due to the limited sample size, the statistical correlation between FIV, CDV and FeLV status of cats in comparison to FeMV antibodies could not be determined. It is, however, important to note that FeMV results obtained using the IFA used in the present study are not attributable to cross-reactivity against either of those feline viruses, especially as might be expected in the case of CDV. The serum samples that were analyzed were collected between 2008 and 2010 and between 2015 and 2016. No differences regarding the seroprevalence of FeMV antibodies were detected between these two periods. It can be concluded that the virus was circulating as early as 2008 in Chile, which is similar to what was reported by Woo *et al.* in Hong Kong [2].

In conclusion, our findings demonstrate a high seroprevalence of FeMV in Chilean free-roaming cats. Our IFA data indicate that FeMV seroprevalence data might be higher than reported in previous serological surveys based solely on the antibody response against a single viral protein. The susceptibility of female and male animals to both FeMV genotypes should be investigated further, as our data suggest possible sex-specific effects regarding the seroprevalence of different FeMV genotypes.

## Electronic supplementary material

Below is the link to the electronic supplementary material.

Supplementary file1 (DOCX 4684 KB)
