# Supplementary material for: High seroprevalence of feline morbilliviruses in free-roaming domestic cats in Chile
Source: Arch Virol. 2020 Nov 20;166(1):281–5. doi: 10.1007/s00705-020-04882-2 (PMC7815546; doi:10.1007/s00705-020-04882-2)
Supplement: Supplementary file 1 — Supplementary file1 (DOCX 4684 KB) [file 705_2020_4882_MOESM1_ESM.docx]

High Seroprevalence of feline Morbilliviruses in free-roaming domestic Cats from Chile

Johannes Busch^1)^, Irene Sacristán^2)^, Aitor Cevidanes^2)^ Javier Millán^3, 4, 5)^, Thomas W. Vahlenkamp^1)^, Constanza Napolitano^6, 7)^, Michael Sieg^1)*^

^1)^ Institute of Virology, Faculty of Veterinary Medicine, University of Leipzig, An den Tierkliniken 29, 04103 Leipzig, Germany

^2)^ PhD Program in Conservation Medicine, Facultad de Ciencias de la Vida, Universidad Andres Bello, República 252, Santiago, Chile

^3)^ Facultad de Ciencias de la Vida, Universidad Andres Bello, República 252, Santiago, Chile

^4)^ Instituto Agroalimentario de Aragón-IA2 (Universidad de Zaragoza-CITA), Miguel Servet 177, 50013 Zaragoza, Spain

^5)^ Fundación ARAID, Avda. de Ranillas, 50018 Zaragoza, Spain

^6)^ Departamento de Ciencias Biológicas y Biodiversidad, Universidad de Los Lagos, Av. Fuchslocher 1305, Osorno, Chile.

^7)^ Instituto de Ecología y Biodiversidad (IEB), Santiago, Chile.

*Correspondence to: [michael.sieg@vetmed.uni-leipzig.de](mailto:michael.sieg@vetmed.uni-leipzig.de); phone: +49 341 / 97 38 204, fax: +49 341 / 97 38219, <https://orcid.org/0000-0003-0799-5787>

Supplementary material

The immunofluorescence assay was evaluated using serum from persistently infected cats. Urine of the same animals which were used for the isolation and propagation of FeMV-1 and FeMV-2, respectively. To further verify the staining results antibodies against either the FeMV-1 N protein and the FeMV-2 P protein were applied. Antibodies were generated as described previously [9], with minor modifications. In brief, the ORF of the N gene of FeMV‑1 (GenBank accession no. MG563820) and the ORF of the P gene of FeMV‑2 (GenBank accession no. MK182089) were amplified by RT‑PCR (SuperScript™ III One-Step RT-PCR System with Platinum™ Taq High Fidelity DNA Polymerase, Thermo Fisher Scientific). PCR-Products were cloned into the bacterial expression vector pGEX4T1 and recombinant plasmids were used for transformation of *E. coli* BL21(DE3). Recombinant proteins were purified via affinity chromatography using Pierce™ Glutathione Agarose (Thermo Fisher Scientific) and used for the immunization of rabbits (N protein) or rats (P protein) by a biotech company (Davids Biotechnologie GmbH, Regensburg, Germany). GST-depleted antibodies were used to stain both FeMV genotypes. Representative images are shown below.


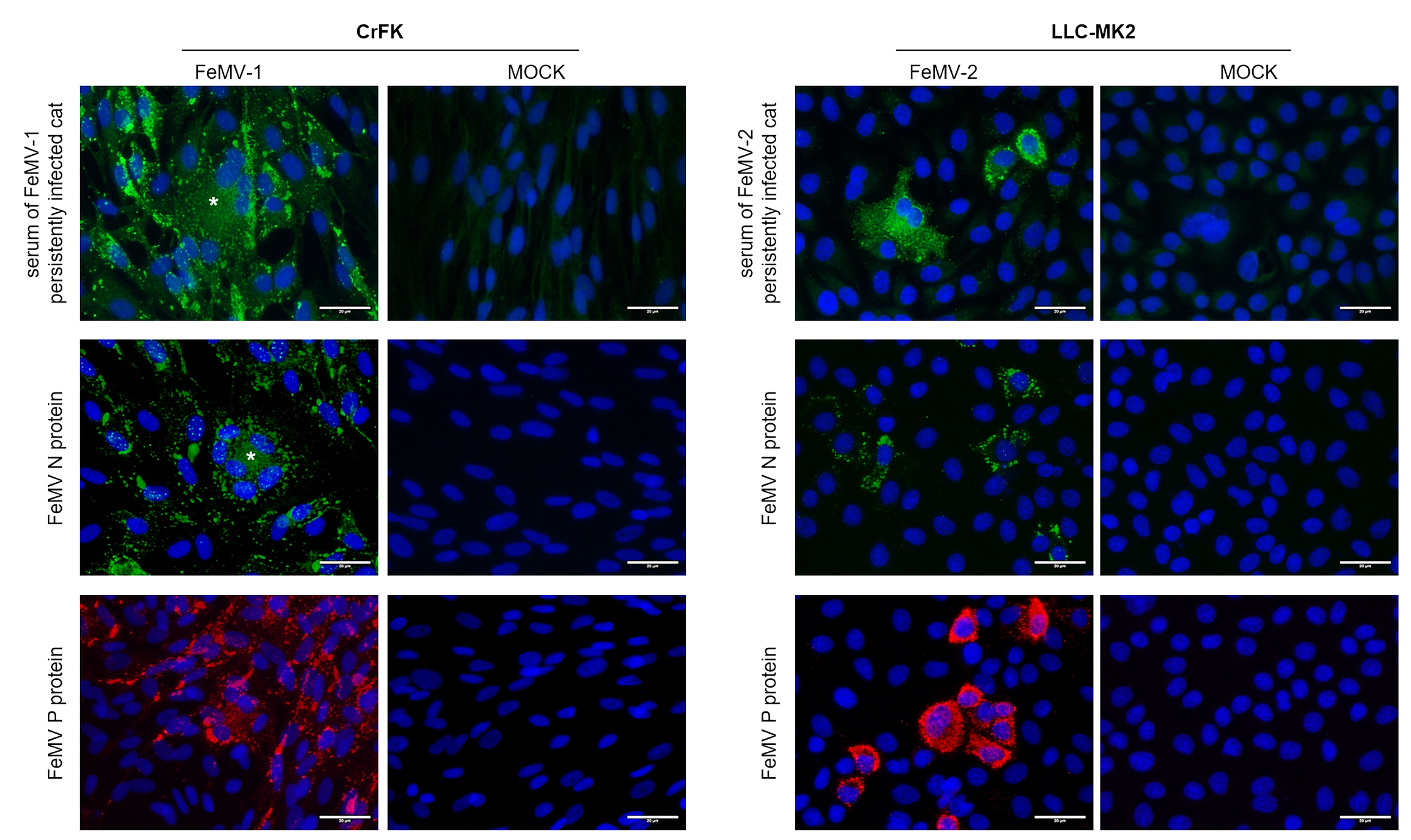


**Supplementary figure 1:** Immunofluorescence assay using cat sera and specific antibodies against the viral N and P protein. Asterisks highlight FeMV-1 induced syncytia formation. Cell nuclei are counter stained with DAPI, shown in blue. Scale bars represent 20 µm.

As depicted in supplementary figure 1, signals detected in infected cells following incubation with sera from FeMV-1 or FeMV-2 persistently infected cats are distinguishable from uninfected cells. Similar staining patterns were observed using FeMV N and P specific antibodies.

Following FeMV-1 infection for five days with a MOI of 0.01, syncytium formation can be observed in a limited number of FeMV-1 positive foci, as it has been reported previously [3].
